# Local Recurrence After Nephron Surgery: What to Do? An Italian Multicentric Registry

**DOI:** 10.3390/cancers17193269

**Published:** 2025-10-09

**Authors:** Angelo Porreca, Filippo Marino, Davide De Marchi, Marco Giampaoli, Daniele D’Agostino, Francesca Simonetti, Antonio Amodeo, Paolo Corsi, Francesco Claps, Alessandro Crestani, Riccardo Bertolo, Alessandro Antonelli, Fabrizio Di Maida, Andrea Minervini, Paolo Parma, Roberto Falabella, Stefano Zaramella, Francesco Greco, Maria Chiara Sighinolfi, Bernardo Rocco, Carmine Sciorio, Antonio Celia, Francesca Romana Prusciano, Pier Paolo Prontera, Gian Maria Busetto, Luca Di Gianfrancesco

**Affiliations:** 1 Department of Urology, Humanitas Gavazzeni, 24125 Bergamo, Italy; angelo.porreca@hunimed.eu (A.P.); filippo.marino@gavazzeni.it (F.M.); davide.demarchi@gavazzeni.it (D.D.M.); marco.giampaoli@gavazzeni.it (M.G.); dott.dagostino@gmail.com (D.D.); francesca.simonetti@gavazzeni.it (F.S.); 2 Medicine and Surgery, Humanitas University, Pieve Emanuele, 20072 Milan, Italy; 3 Department of Urology, Veneto Institute of Oncology IOV—IRCCS, 35128 Padua, Italy; antonio.amodeo@iov.veneto.it (A.A.); paolo.corsi@iov.veneto.it (P.C.); francesco.claps@iov.veneto.it (F.C.); 4 Azienda Sanitaria Universitaria Friuli Centrale, 33100 Udine, Italy; alessandro.crest@gmail.com; 5 Azienda Ospedaliera Universitaria Integrata Verona, 37126 Verona, Italy; riccardobertolo@hotmail.it (R.B.); alessandro.antonelli@univr.it (A.A.); 6 Azienda Ospedialiero-Universitaria Careggi, 50134 Firenze, Italy; fabrizio.dimaida@unifi.it (F.D.M.); andrea.minervini@unifi.it (A.M.); 7 Azienda Ospedaliera Carlo Poma, 46100 Mantova, Italy; paolo.parma@asst-mantova.it; 8 Ospedale S. Carlo, 85100 Potenza, Italy; rfalabella@libero.it; 9 Ospedale degli Infermi, 13875 Biella, Italy; stefano.zaramella@aslbi.piemonte.it; 10 Policlinico San Pietro, Gruppo San Donato, Ponte San Pietro, 24036 Bergamo, Italy; francesco_greco@ymail.com; 11 Agostino Gemelli University Polyclinic, 00136 Roma, Italy; sighinolfic@gmail.com (M.C.S.); bernardo.rocco@gmail.com (B.R.); 12 Clinica San Martino, 23864 Lecco, Italy; carmine.sciorio@gmail.com; 13 Ospedale San Bassiano, 36061 Bassano del Grappa, Italy; antonio.celia@aulss7.veneto.it; 14 Ospedale SS. Annunziata, 74121 Taranto, Italy; francescaromana.prusciano@asl.taranto.it (F.R.P.); pierpaolo.prontera@asl.taranto.it (P.P.P.); 15 Ospedali Riuniti di Foggia, Università di Foggia, 71122 Foggia, Italy; gianmaria.busetto@unifg.it

**Keywords:** local recurrence, partial nephrectomy, radical nephrectomy, positive surgical margin, prognostic factor, review, renal cell carcinoma

## Abstract

Introduction and Objectives: Local recurrence (LR) in patients treated with surgery for renal cell carcinoma (RCC) remains a significant clinical challenge that requires thorough investigation. Our study aimed to identify the relative risk factors and explore the optimal clinical management of LR. Materials and Methods: We conducted a non-randomized, observational, retrospective multicentric registry involving multiple Italian urological centers. We included patients treated with surgery (either nephron-sparing or radical nephrectomy) who later developed LR, defined as recurrence in the ipsilateral kidney or renal fossa. Patients with hereditary syndromes or metastatic disease at the time of LR diagnosis were excluded. Results: We reported 135 cases of LR with the following characteristics: most primary lesions were monofocal (85.7%), with a median size of 42 mm (23–53), the median R.E.N.A.L. score was 7 (6–8), and the median Padua score was 7 (6–9). Patients were treated with robot-assisted techniques in 59% of cases, laparoscopic surgery in 32.4%, and open surgery in 8.6%. Nephron-sparing surgery was performed in 75.2% of cases. Ischemia occurred in 61% of the cases, with a median ischemia time of 21 min (15.5–24). Intraoperative complications occurred in 3.8% of cases, while postoperative complications were reported in 13.8%, all of which were grade ≤3 according to the Clavien–Dindo classification. The primary tumors were pT1a in 43.5% of cases, pT1b in 26.3%, pT2 in 14.7% and pT3 in 15.5%. Histologically, 84% of cases were clear cell, 11.3% papillary type 1 or 2, and 3.7% chromophobe. Sarcomatoid/rhabdoid variants were present in 10.5% of cases. The median rate of LR was 1.3% (range 0.2–3.6), while the median time to LR was 18 months (12–39). LR occurred in the ipsilateral kidney in 70.5% of cases and in the ipsilateral renal fossa in 29.5%. The median rate of PSM in LR cases at initial surgery was 2.4% (range 0–4.3), while the median rate of negative surgical margin (NSM) in LR cases at initial surgery was 0.1 (0–0.3). Following LR diagnosis, most patients (49.2%) underwent surgery, 29.1% received cryoablation or radiotherapy, 17.1% received systemic treatment alone, and 4.6% followed a watchful waiting/active surveillance approach. At a median follow-up of 62 months, the highest oncological control in terms of 5-year cancer-specific survival and overall survival rates was achieved in surgically treated patients. The PSM, the histological variant, and their combination were found to be independent variables correlated with the occurrence of LR, with relative risks of 3.62, 2.71, and 8.12, respectively. Conclusions: LR after nephron-sparing or radical nephrectomy represents a significant clinical dilemma. Known risk factors are not always sufficient to predict recurrence, emphasizing the necessity of consistent radiological follow-up per guideline recommendations. Early detection of recurrence and a multidisciplinary approach involving expert centers are crucial for optimizing patient outcomes.

## Simple Summary

Patients who undergo surgery for kidney cancer could, in a small but important number of cases, see the cancer return near the original site. Because it is hard to predict who is at risk and which treatment works best, we studied real-world cases from several Italian centers. We analyzed 135 patients who experienced local recurrence after undergoing partial or complete kidney removal. Recurrence usually appeared within about 18 months. It was more likely when the first operation left cancer cells at the cut edge and when the cancer had more aggressive cell types. Treating the recurrence with surgery achieved the best cancer control, while kidney function was generally preserved across approaches. These findings can help refine follow-up schedules, identify higher-risk patients, and guide multidisciplinary care, informing future research and personalized treatment strategies.

## 1. Introduction

Local recurrence (LR) after renal surgery for renal cell carcinoma (RCC) represents a significant clinical challenge and remains a focal point of ongoing investigation. LR is a critical concern for patients who have undergone partial nephrectomy (PN) or radical nephrectomy (RN), given its implications for patient outcomes and the necessity for thorough post-operative surveillance or treatment [1]. Despite advancements in surgical techniques and an improved understanding of RCC biology, the risk factors and optimal management strategies for LR are still not fully understood.

International guidelines currently recommend PN as the standard of care for clinical T1a and many T1b RCC cases due to its potential to preserve renal function while achieving oncological control [2]. However, this approach has its limitations. One of the discussed risk factors for LR is the presence of positive surgical margins (PSM), which can occur in 0.1–10.7% of PN cases [3]. The relationship between PSM and LR is a subject of ongoing debate and controversy. Some studies suggest that PSM is a strong predictor of LR and can affect the risk of metastasis and cancer-specific survival, while others do not find a significant correlation [4,5,6,7]. However, the prevailing view is that patients with PSM are at increased risk of disease recurrence, especially with highly malignant tumors [4,8]. Traditionally, LR has been attributed to incomplete tumor removal, multifocality, or the development of new tumors in areas other than the resection bed [1].

The definition of LR varies widely in the literature. However, the most accepted definition is the presence of recurrence at the tumor bed or near the original tumor site in the ipsilateral kidney or renal fossa, as determined by radiological imaging [9]. The rate of LR following PN has been demonstrated to be less than 5%, which is comparable to the rates observed after RN [10]. In a multi-institutional French study analyzing 110 patients who underwent robot-assisted partial nephrectomy (RAPN), the rate of LR was 2.7% [11]. In a long-term study by Bertolo et al. involving patients who underwent RAPN, the cumulative incidence of LR was 3.24% and 4.57% at 5 and 7 years, respectively [12]. A meta-analysis by Minervini et al. involving 11,282 patients found that the prevalence of LR after PN was 2.3% [1]. According to the literature, the majority of LR following surgery occur within the initial two years, with median follow-up periods varying from 24 to 162 months [9].

Current guidelines emphasize the importance of follow-up imaging to detect recurrences early, but the optimal management strategy for LR remains unclear [13]. The decision-making process is complex and depends on multiple factors, including tumor biology, anatomical location of the recurrence, patient comorbidities, and previous treatment modalities.

The literature, whether from single-center or multicenter studies, presents small patient cohorts experiencing disease recurrence, which complicates drawing definitive conclusions. Hence, the debate remains ongoing. To address this clinical challenge, the “Italian Group for Advanced Laparo-Endoscopic Surgery (AGILE Group)” non-profit foundation has designed a multicentric, retrospective registry to investigate LR after renal surgery. This study aimed to identify any risk factors associated with LR, evaluate the association between PSM and LR, evaluate the time to LR, and assess survival rates, thereby evaluating the efficacy of different clinical management strategies by analyzing treatment patterns and providing insights into the best clinical management approaches. The study included patients from various Italian urological centers who had experienced LR following either nephron-sparing or radical nephrectomy. In summary, the AGILE Group’s efforts aimed to enhance patient care through evidence-based strategies and collaborative research, significantly advancing the understanding and management of LR in renal cancer.

## 2. Materials and Methods

### 2.1. Study Design

This was an observational, retrospective, multicenter, cohort study in cooperation with the AGILE Group, which involved a group of urologists from various urological Departments of Italy who routinely perform laparoscopic and robotic surgeries. This registry was intended to be prospectively maintained.

All demographic, clinical, pathological, and surgical variables were obtained from a registry that was prospectively maintained by each participating center and subsequently reconstructed retrospectively for the present study in a retrospective observational cohort study.

### 2.2. Endpoints

The primary endpoint was to evaluate the local recurrence rate and the time to local recurrence after surgery for RCC.

The secondary objective was to determine other survival and functional outcomes and perform a prognostic factors analysis.

Secondary endpoints evaluated were:

- Overall Survival (OS): the time between surgery and the date of death or last follow-up.
- Disease Free-Survival (DFS): interval between surgery and the occurrence of LR or cancer-related death; patients who are alive and disease-free at the last follow-up are censored.
- Progression Free-Survival (PFS): the time between surgery and the first event of clinically documented recurrence or death from RCC, censoring the case with recurrence
- Cancer-specific survival (CSS): the time of surgery to the date of cancer-related death, with patients who do not die from cancer being censored at the last follow-up.
- Association between prognostic factors (positive surgical margins and other significant covariates) and local recurrence.
- Evaluation of functional outcomes with creatinine values and estimated glomerular filtration (eGFR) rate.

### 2.3. Study Participants

The registry collected data on patients who underwent kidney surgery for RCC, including both RN and PN, and experienced a LR following the initial surgery. We included patients with a clinical or histologically confirmed diagnosis of LR of renal tumor occurring between 1 January 2015 and 31 December 2024, and treated at one of the participating centers. All included patients represented consecutive cases of local recurrence across participating centers. Data were obtained from institutional registries that were prospectively maintained and subsequently reconstructed retrospectively for the present analysis. Case identification was based on pathology reports, operative notes, and follow-up records, and all entries were validated independently by each center’s clinical team before integration into the pooled dataset.

### 2.4. Definition of Local Recurrence

For the purpose of this study, LR was defined in a uniform manner as the reappearance of renal cell carcinoma in the ipsilateral kidney or in the ipsilateral renal fossa after initial nephron-sparing surgery, confirmed by imaging and/or histology. Recurrences at distant sites (contralateral kidney, lymph nodes, or extra-renal metastases) were not included in this definition. This operational definition was applied consistently across all centers and throughout the analysis.

### 2.5. Inclusion/Exclusion Criteria

Patient inclusion criteria:

- Male or female;
- Age ≥ 18 years;
- Patients treated with pPN or RN for histologically confirmed RCC;
- Patients who experienced LR occurring in the ipsilateral kidney or renal fossa;
- Written informed consent signed;
- Minimum follow-up of 24 months from surgery.

Patient exclusion criteria:

- Genetic predisposition to kidney cancer to prevent the inclusion of cases with synchronous or metachronous tumors;
- Metastatic disease, including lymph node involvement or distant localizations at the time of local recurrence.

### 2.6. Participating Centers

**Figure 1:**
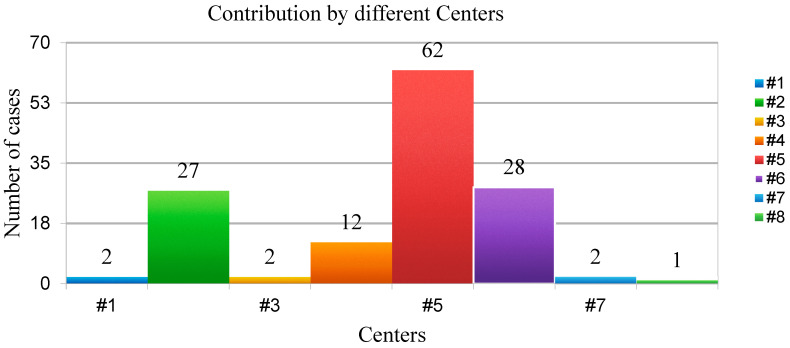
Contribution by different centers.

All members of the AGILE Group experienced in renal surgery were invited to voluntarily participate in this project, with no additional funding provided to the participating centers. Among all, eight Italian Urological Departments have decided to participate and provide data on patients with LR following renal surgery. The participating centers were as follows: Department of Urology, Humanitas Gavazzeni, Bergamo; Department of Oncological Urology, Veneto Institute of Oncology (IOV)—IRCCS, Padua; Department of Urology, AOU Careggi, Florence; Department of Clinical Urology, Biella Hospital, Biella; Galliera Hospital, Genova; Urology Unit, ASST Santi Paolo and Carlo, Milan; Hospital Bassiano, Bassano; Urology Unit, San Carlo, Potenza (Figure 1).

All demographic, clinical, pathological, and surgical variables were obtained from a registry that was prospectively maintained by each participating center and subsequently reconstructed retrospectively for the present study in a retrospective observational cohort study.

### 2.7. Enrolment of Patients

Patient recruitment began at the participating centers by retrospectively evaluating case histories starting from 1 January 2015, to identify patients who had experienced an LR. All potential participants (directly treated for the initial diagnosis of renal tumor by the specific center, or patient cases discussed during multidisciplinary meetings conducted in a specific center participating in the study) were invited for a visit and provided with an informational brochure detailing the study. Relevant data regarding the first renal tumor surgery and subsequent treatments for LR were collected from the hospital’s internal systems and medical records. Following this, the patient began prospective follow-up.

Recruitment continued until 31 December 2024, and all patients who developed an LR and met the inclusion criteria were included and started prospective follow-up.

All procedures and patient management were conducted according to clinical practice standards and international guidelines, and data were stored at the participating centers following the national laws.

### 2.8. Follow-Up

The dataset was organized with fields to input follow-up data at 1, 3, 6, and 12 months after the initial surgery, followed by annual intervals thereafter, according to international guidelines [13]. This structure enabled precise identification of the timing of LR for each patient and evaluation of the chosen treatment approach. At each follow-up point, data regarding creatinine and eGFR were required. Once LR occurs, the patients continued to be monitored prospectively with the same follow-up schedule at 1, 3, 6, 12 months, and then annually.

### 2.9. Variables of Interest

At each center, the designated physician ensured the collection of pertinent variables of interest and required follow-up data. Specifically, the following data were collected:

- patient data: gender and date of birth
- preoperative data: date of the first renal surgery for RCC, age at diagnosis, smoking status, American Society of Anesthesiologists (ASA) score, Carlson comorbidity score [14]. Preoperative clinical tumor stage based on computed tomography (CT) or magnetic resonance imaging (MRI), focality and number of renal lesions, size of the largest lesion (mm), preoperative eGFR, RENAL Nephrometry score, and PADUA score.
- perioperative data: data on the first renal surgical procedure were categorized according to the technique used for RN or PN (open, laparoscopic, or robotic-assisted), total time of surgery (minutes), and estimated blood loss (EBL) (mL), off-clamp or on-clamp procedure, eventual ischemia time (minutes), intraoperative and postoperative Clavien–Dindo complications [15]
- postoperative data: assessment of postoperative grading and stage of the renal tumor was performed according to international criteria [13] including pathological tumor-node-metastasis (pTNM) stage, positive surgical margins, histology and eventually variants/aspects. Histological variants were defined as renal cell carcinoma (RCC) subtypes other than conventional clear cell carcinoma, including papillary RCC, chromophobe RCC, and cases with sarcomatoid or rhabdoid differentiation. For analytical purposes, these subtypes were grouped together as “histological variants” given their recognized association with more aggressive clinical behavior. The median rate of PSM referred to the central tendency, or middle value, of reported PSM rates across the series of surgical cohorts. A positive surgical margin is defined as the presence of tumor cells at the inked resection margin of the specimen, indicating that malignant tissue may have been left behind, which is an important quality indicator in oncological surgery. Reporting the median rather than the mean minimizes the influence of outliers and provides a more robust measure of the typical margin status outcome in a given population. The median rate of LR was defined as the central value of reported recurrence rates across multiple patient cohorts, representing the proportion of individuals who experience tumor regrowth at or near the original surgical site during follow-up. Local recurrence reflected either residual microscopic disease left behind after resection or new tumor development within the same anatomical region, and its rate serves as an important indicator of both surgical completeness and underlying tumor biology. The use of the median instead of the mean is preferred in pooled analyses, as it reduces the impact of extreme values or outliers, thereby providing a more stable and representative measure of typical recurrence risk. These parameters are frequently reported in oncological surgery to assess long-term oncologic control, guide comparisons between different surgical techniques, and inform patient counseling and follow-up strategies.
- follow-up and outcomes: follow-up data were obtained from prospectively maintained institutional registries that systematically recorded surveillance visits and outcomes and were subsequently reconstructed retrospectively for the present analysis. In all centers, patients were monitored according to a structured schedule after the initial renal surgery, with evaluations typically performed at 1, 3, 6, and 12 months, and annually thereafter. At each follow-up, renal function (including eGFR values), imaging findings, and oncological status were documented. In the event of local recurrence, the date, time to recurrence, and management strategy (watchful waiting, surgery, systemic therapy, radiotherapy, or cryoablation) were recorded in the registry. Mortality data, including date and cause of death, were also prospectively documented, enabling calculation of OS and CSS. This approach ensures that the follow-up information presented in this study reflects systematically collected clinical data rather than assumptions derived solely from guideline-based protocols.

### 2.10. Statistical Analysis

Data were cleaned and reviewed for discrepancies by a statistician prior to analysis. If any data was missing, the responsible physician was contacted to review the medical records and data sheet for the missing information. The characteristics of patients with missing data were examined, and if necessary, sensitivity analysis or multiple imputation was employed to determine the impact of missing data on the results.

Data were presented as frequency (percent) for categorical variables, as mean ± standard deviation (SD) for variables with a normal distribution, and as median and interquartile range for variables with a non-normal distribution. Student’s *t*-test or Mann–Whitney U test was used to compare continuous variables, while the Pearson chi-square test was employed to analyze categorical variables.

OS was defined as the time between surgery and the date of death or last follow-up. CCS was measured from the time of surgery to the date of cancer-related death, with patients who did not die from cancer being censored at the last follow-up. Survival rates were estimated using the Kaplan–Meier method with their 95% confidence intervals.

After performing a 1:2 matched analysis with consecutive patients from the same registry who did not develop LR, we constructed a comparative cohort. Matching was carried out on key baseline variables considered most relevant to recurrence risk, including age at surgery (±5 years), pathological tumor stage (pT1 vs. ≥pT2), surgical approach (open, laparoscopic, robotic), and surgical type (radical vs. nephron-sparing). Covariate balance after matching was assessed using standardized mean differences, with all matching variables achieving SMD < 0.1, confirming adequate balance.

Subsequently, we evaluated the relative risk of LR and performed logistic regression analyses, considering the occurrence of LR as the dependent variable (intercept *y*) and the following predictors: positive surgical margins (PSM), histological variants, and their combination. These variables were selected a priori based on their biological and clinical relevance to recurrence. To avoid overfitting, given the limited number of LR events in some strata, the regression model was restricted to these predefined predictors. Exploratory models, including additional variables such as tumor grade and surgical approach, were also tested and did not materially alter the direction or statistical significance of the associations. Odds ratios (OR) with 95% confidence intervals (95% CI) and corresponding *p* values were reported.

Univariable comparisons are presented as incidence proportions and relative risks (RR) with 95% confidence intervals (CI). For comparisons with expected cell counts ≤5, we used Fisher’s exact test. To obtain adjusted estimates, we fitted a multivariable logistic regression model including histological variant status, positive surgical margins (PSM), and their combination. Logistic regression coefficients are presented as adjusted odds ratios (OR) with 95% CI. Because several subgroup analyses included small numbers of events, we report absolute event counts and incidence proportions alongside relative measures to improve interpretability. All tests were two-sided with α = 0.05.

In this study, our primary objective was to identify independent variables associated with the occurrence of local recurrence, rather than to model the time to recurrence. For this reason, we selected logistic regression, which is specifically suited for binary outcomes and provides interpretable odds ratios that quantify the strength of association between surgical and pathological features and the risk of recurrence. Although follow-up was sufficiently long, the number of recurrence events within some subgroups—particularly patients with histological variants—was relatively small. Under such conditions, Cox proportional hazards models may yield unstable estimates and are sensitive to violations of the proportional hazards assumption. Logistic regression was therefore statistically more robust in our dataset. An additional advantage was the possibility to integrate absolute event counts and incidence proportions alongside odds ratios, thereby increasing transparency and interpretability, particularly in light of some imprecise univariable risk ratios. We performed survival analyses using Kaplan–Meier estimates and log-rank tests to assess oncological outcomes by treatment modality. However, the multivariable analysis presented in Table 2 and Table 3 was specifically designed to evaluate predictors of recurrence occurrence, not hazard over time, and logistic regression was therefore the most appropriate tool for this aim. While Cox regression could certainly provide complementary insights into time-to-recurrence in larger series, we considered logistic regression to be the most robust and transparent approach for the present study.

### 2.11. Missing Data Handling

All demographic, clinical, pathological, and surgical variables were prospectively collected and retrospectively analyzed. The completeness of the dataset was high, with missing data observed in <5% of cases across all reported parameters. When missingness occurred (e.g., ASA score, AACII score, smoking history, or pre-operative eGFR), patients were excluded only from the specific analysis requiring the unavailable variable, while remaining eligible for all other analyses. Tumor- and surgery-related variables (e.g., R.E.N.A.L. score, Padua score, ischemia time) with occasional gaps were similarly handled without data substitution, and the frequency of missingness was explicitly reported in descriptive statistics. To ensure robustness, sensitivity analyses confirmed that the exclusion of incomplete cases did not materially alter the direction or statistical significance of the outcomes. Because the extent of missing data was minimal and no systematic patterns suggesting bias were identified, advanced imputation methods were deemed unnecessary. This conservative approach strengthens the validity of the results and aligns with STROBE recommendations, ensuring that the associations observed between surgical, pathological, and oncological factors and local recurrence are unlikely to be explained by missing data.

**Responsibilities of the Scientific and Steering Committees**: The Steering Committee is respon-sible for overseeing the planning and execution of the registry. Their responsibilities include approving the participating centers and the corresponding physicians in charge of data collec-tion, conducting quality control of the data, guiding and suggesting changes to meet the pro-ject’s objectives, and analyzing and revising the results for presentation at conferences and pub-lication in scientific journals. The Scientific Committee’s role is to guide the preparation of scien-tific papers and presentations of results at conferences, in consultation with the Steering Com-mittee. They ensure adherence to authorship eligibility guidelines for all publications.

**Ethical considerations**: Prior to initiation of a study site, approval from the appropriate EC will be obtained in accordance with applicable country-specific regulatory requirements. The study will be conducted in accordance with applicable subject privacy requirements. All participants are required to provide written informed consent. Data collection will be conducted in accord-ance with the World Medical Association Declaration of Helsinki. The standard of care remains consistent with that provided according to international guidelines for patients participating in the study.

**Consent for publication**: We obtained written informed consent to publish, but the written con-sent itself was held by the investigators themselves, in the patient’s hospital record.

## 3. Results

### 3.1. Patient and Tumor Characteristics

A total of 135 patients with LR were analyzed (Table 1). The median age at LR diagnosis was 64 years (58–70), with a predominance of males (73.3%). The median ASA score was 2 (2–3), and the median AACII score was 4 (4–5). A history of smoking was reported in 50.4% of patients.

The median pre-operative eGFR was 67.4 mL/min/1.73 m^2^ (range 38–98).

The primary tumors were monofocal in 85.7% of cases, with a median size of 42 mm (23–53).

The median R.E.N.A.L. score was 7 (6–8), and the median Padua score was 7 (6–9).

In terms of pathological staging, 43.5% of tumors were pT1a, 26.3% were pT1b, 14.7% were pT2, and 15.5% were pT3.

The predominant histological subtype was clear cell RCC (84%), followed by papillary RCC (11.3%) and chromophobe RCC (3.7%). Sarcomatoid/rhabdoid differentiation was noted in 10.5% of cases.

When stratifying by histological subtype, tumors with variants other than conventional clear cell RCC (papillary, chromophobe, and cases with sarcomatoid/rhabdoid differentiation) demonstrated a higher proportion of local recurrence compared to clear cell tumors. Similarly, the presence of positive surgical margins at initial surgery was associated with an increased risk of recurrence. Patients presenting with both a histological variant and positive surgical margins exhibited the highest recurrence rates, although the number of events was limited. Due to the small sample size in each variant subgroup, these findings should be considered exploratory and hypothesis-generating rather than definitive evidence of subtype-specific risk.

### 3.2. Surgical and Oncological Outcomes

Nephron-sparing surgery was performed in 75.2% of cases, with a robotic approach being the most common (59%), followed by laparoscopic (32.4%) and open surgery (8.6%).

The median surgery duration was 140 min (120–182), and the median estimated blood loss was 200 mL (180–300). Ischemia was necessary in 61% of cases, with a median ischemia time of 21 min (15.5–24).

The median rate of PSM in LR cases at initial surgery was 2.4% (range 0–4.3), while the median rate of negative surgical margin (NSM) in LR cases at initial surgery was 0.1 (0–0.3).

Intraoperative complications occurred in 3.8% of cases, while postoperative complications were observed in 13.8%, all classified as ≤3 according to the Clavien–Dindo classification.

We did not report cases of re-operation.

### 3.3. Local Recurrence Patterns and Management

The median rate of LR was 1.3% (range 0.2–3.6)

The median time to LR was 18 months (12–39).

Recurrence was observed in the ipsilateral kidney in 70.5% of cases and in the renal fossa in 29.5% of cases.

**Figure 2:**
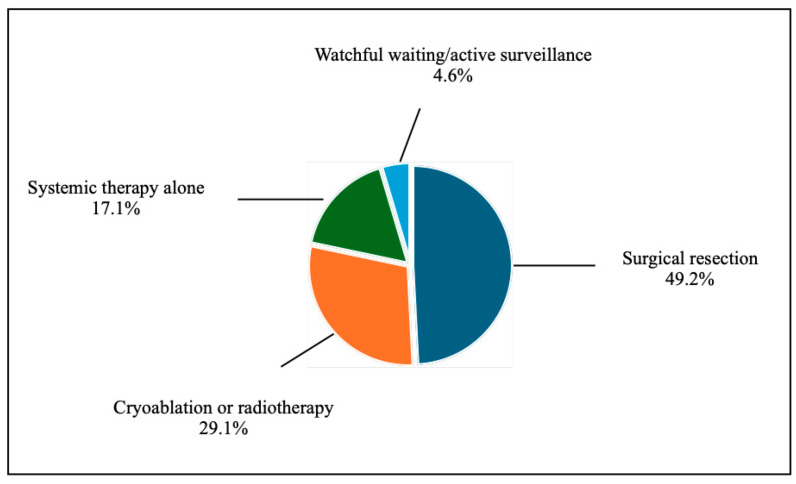
Management of LR.

Treatment strategies for LR were diverse (Figure 2):

- Surgical resection: 49.2%
- Cryoablation or radiotherapy: 29.1%
- Systemic therapy alone: 17.1%
- Watchful waiting/active surveillance: 4.6%

### 3.4. Renal Function Outcomes

Serial renal function assessment was available for all patients. The median preoperative eGFR at initial surgery was 67.4 mL/min/1.73 m^2^ (range 38–98), and at the time of local recurrence diagnosis, the median eGFR was 64.1 mL/min/1.73 m^2^ (range 36–95), corresponding to a median decline of 3.3 mL/min/1.73 m^2^ (−4.9%, *p* = 0.08 vs. baseline). Following treatment of recurrence, the median eGFR at the last follow-up was 62.7 mL/min/1.73 m^2^ (range 34–92), representing a cumulative reduction of 6.9% compared with baseline (*p* = 0.04). Patients undergoing repeat nephron-sparing surgery (*n* = 101) demonstrated a median decline of 5.8 mL/min/1.73 m^2^ (−8.6%), which reached statistical significance compared to baseline (*p* = 0.03). In the subgroup managed with cryoablation or radiotherapy (*n* = 39), renal function was better preserved, with a non-significant median decrease of 1.9 mL/min/1.73 m^2^ (−3.2%, *p* = 0.21). Patients treated with systemic therapy (*n* = 23) showed greater variability, with a median eGFR decline of 7.6 mL/min/1.73 m^2^ (−11.3%, *p* = 0.02), likely reflecting treatment-related nephrotoxicity. Comparative analysis confirmed that renal function decline was significantly greater in patients receiving systemic therapy compared to those managed with cryoablation/radiotherapy (*p* = 0.01), while the difference between repeat nephron-sparing surgery and cryoablation did not reach statistical significance (*p* = 0.09). Importantly, no patient in our series developed end-stage renal disease or required dialysis during follow-up. These findings indicate that renal function remained largely preserved across different LR management strategies, with superior outcomes in patients undergoing nephron-sparing or minimally invasive approaches.

### 3.5. Correlation

We reported that the presence of PSM, the histological variant, and their combination represented independent variables of correlation with the occurrence of LR (Table 2); subsequently, the risk of LR was higher in the case of the combination of the presence of PSM and the histological variant (Table 3).

**Table 2:** Logistic regression.

	Regression Coefficients	Standard Error	Stat t	OR	95%CI	*p* Value
Intercept *	0.315	0.024	13.268	1.37	1.31–1.44	0.000
Histological variants	0.385	0.107	3.606	1.47	1.19–1.81	0.001
PSM	0.311	0.144	2.162	1.36	1.03–1.81	0.031
Combination (PSM + Histological variants)	0.472	0.211	2.230	1.60	1.06–2.43	0.026

* Intercept: occurrence of LR.

**Table 3:** Relative risk (retrospective evaluation).

	RR	Confidence Interval		*p* Value
		Lower 95%	Upper 95%	
PSM	3.62	1.04	2.60	0.03
Histological variants	2.71	1.20	6.15	0.01
Combination (PSM + Histological variants)	8.12	0.90	73.4	0.02

Some univariable RRs in Table 3 seemed to be imprecise. For example, RR 8.12 had a 95% CI of 0.90–73.4, reflecting few events in the compared strata and a consequent large standard error of the log-risk (approximate SE of ln(RR) ≈ 1.12). To increase transparency, we have added absolute event counts and incidence proportions to Table 3 and performed exact tests where appropriate. In addition, multivariable logistic regression confirmed independent associations between local recurrence and both histological variants and positive surgical margins: histological variants, adjusted OR 1.47 (95% CI 1.19–1.81), *p* = 0.001; PSM, adjusted OR 1.36 (95% CI 1.03–1.81), *p* = 0.031; combination (PSM + variant), adjusted OR 1.60 (95% CI 1.06–2.43), *p* = 0.026. These adjusted estimates indicated a consistent direction of effect despite imprecision in some univariable RRs.

### 3.6. Follow-Up

The median follow-up duration for the entire cohort was 62 months (interquartile range [IQR] 41–84 months). Follow-up data were available for 132 of 135 patients, corresponding to a follow-up rate of 97.8%. Only three patients were lost to follow-up after a median of 28 months (range 24–33). At 3 years, follow-up completeness was 96.3%, and at 5 years, it remained 92.6%. Survival analyses were therefore conducted on a near-complete dataset, ensuring the robustness of oncological and functional outcome estimates.

**Figure 3:**
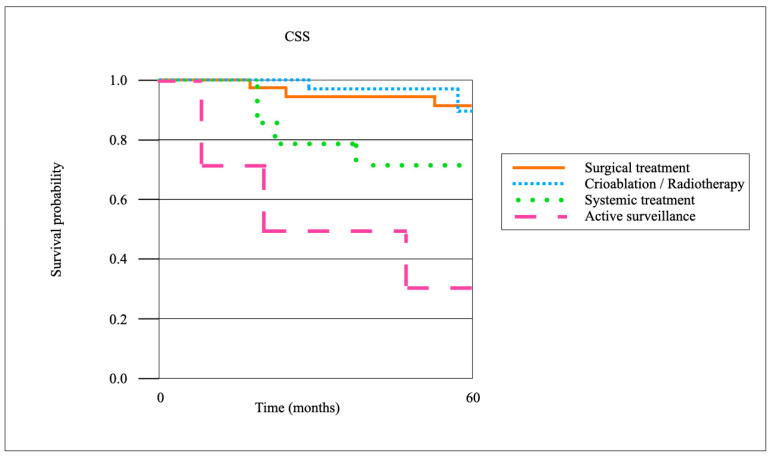
Kaplan–Meier survival curves on CSS.

**Figure 4:**
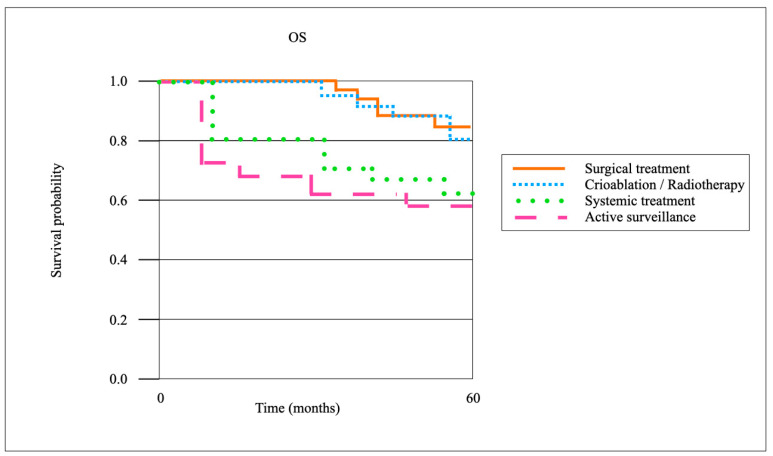
Kaplan–Meier survival curves on OS.

When stratified by treatment modality for local recurrence, surgical resection achieved the highest oncological control rates. The 3-year local recurrence-free survival (LRFS) after surgical resection (*n* = 66) was 86.4%, compared with 72.1% after cryoablation or radiotherapy (*n* = 39), and 41.7% with systemic therapy alone (*n* = 23) (*p* < 0.01). The CSS at 5 years was 92.7% in the surgical cohort, 88.1% in the cryoablation/radiotherapy group, and 71.3% in patients managed with systemic therapy (*p* = 0.03). The OS at 5 years was 85.5%, 80.2%, and 63.9%, respectively (*p* = 0.04). Patients managed with active surveillance (*n* = 7) demonstrated inferior outcomes, with a 3-year LRFS of 28.6% and a 5-year OS of 57.1%. Pairwise comparisons confirmed significantly improved LR control after surgical resection compared to cryoablation/radiotherapy (*p* = 0.02), although CSS differences between these two groups did not reach statistical significance (*p* = 0.18) (Figure 3 and Figure 4).

## 4. Discussion

The LR following surgical treatment for RCC remains an important clinical challenge. Despite advances in imaging and surgical techniques, recurrence patterns are not entirely predictable, and the risk factors identified in this study suggest that tumor size, histology, and surgical approach may play a role.

Our multicentric registry aimed to provide valuable insights into the risk factors, incidence, and management of LR. These findings aligned with previous literature.

Our study aimed to identify key risk factors associated with LR, including tumor characteristics, surgical approach, and patient demographics. Consistent with previous findings, we observed that tumors with higher complexity scores (R.E.N.A.L. and Padua scores) were more likely to recur. Similarly, Marchiñena et al. [16] and Radfar et al. [17] found that patients with positive PSM and high Fuhrman grade tumors had an increased risk of LR.

The correlation between surgical margins and LR remains debated in the literature. Studies report varying PSM rates, from 0% to 34.4%, with corresponding LR rates of 0% to 9.1% [18,19,20]. In our cohort, LR occurred in 70.5% of cases in the ipsilateral kidney and in 29.5% of cases in the renal fossa, which is consistent with reports by Khalifeh et al. [8] and Çinar et al. [21].

The surgical technique used for primary tumor resection plays a crucial role in LR. Our findings suggest that robot-assisted surgery was the most frequently used approach (59%), followed by laparoscopic (32.4%) and open surgery (8.6%). A similar distribution was observed in previous studies, where minimally invasive techniques demonstrated favorable perioperative outcomes but did not eliminate the risk of LR. Perioperative factors, such as ischemia time and blood loss, were within expected ranges, consistent with reports by Morrone et al. [22], who found an association between prolonged ischemia time and PSM.

We found that the median time to LR was 18 months, aligning with Radfar et al. [17], who reported a mean recurrence time of 9 months, and Tellini et al. [7], who found a median of 43 months for PSM and 56 months for NSM patients. Most LR cases occur within the first two years post-surgery, emphasizing the need for intensive surveillance during this period.

Survival outcomes remain a topic of debate. Some studies suggest that PSM does not significantly impact OS [17], while others indicate poorer recurrence-free survival and metastasis-free survival in PSM patients [8]. Our findings reinforced the importance of individualized follow-up and intervention strategies to optimize long-term oncologic outcomes.

Our analysis confirmed that PSM and histological variants were independently associated with LR, with the combination of these factors conferring the highest risk. These results align with multiple series demonstrating that PSM increases the likelihood of recurrence, particularly in patients with aggressive pathological features [23,24]. However, the prognostic value of PSM remains debated, with some studies suggesting that it has a limited long-term impact on survival once tumor stage and grade are accounted for [25,26]. Our findings reinforce the interpretation that PSM represents a surrogate for technical and biological complexity, and its impact is most clinically relevant when combined with unfavorable histology.

The strong association between non-clear cell RCC subtypes (papillary, chromophobe) or dedifferentiated histologies (sarcomatoid/rhabdoid) and recurrence risk is consistent with prior reports that highlight the aggressive biological potential of these entities [9,27]. Notably, we observed that patients with both PSM and variant histology exhibited the highest recurrence rates, although the small subgroup size limits definitive conclusions. Nonetheless, this finding underscores the importance of integrating pathological subtype into recurrence risk stratification.

In line with earlier studies, we also found that tumor size and anatomical complexity (median R.E.N.A.L. 7, Padua 7) were high in our LR cohort. Prior literature demonstrates that larger, central, or sinus-adjacent tumors confer elevated recurrence risk [23,27], which likely reflects both biological aggressiveness and the technical challenges of complete tumor clearance.

The majority of patients had undergone robot-assisted NSS, consistent with contemporary practice trends. Our reported PSM rate of 2.4% is at the lower range of published series [24,25], reflecting surgical expertise, yet underscores that even in expert hands, PSM cannot be entirely avoided.

The overall median LR rate of 1.3% in our cohort is comparable to previously published ranges (0.5–5%) [9,28], confirming the rarity of this event but also its clinical relevance given the need for tailored management. The median time to LR of 18 months is consistent with the typical window of recurrence described in the literature, though late events remain possible, reinforcing the importance of prolonged follow-up [28].

The management of LR remains heterogeneous, with surgical reintervention being the most common approach (49.5%). Alternative treatments such as cryoablation or radiotherapy were used in 32.3% of cases, while systemic therapies were reserved for 19.1% of patients. These findings align with EAU guidelines, which recommend local treatment when feasible and close surveillance for select patients [13].

Active surveillance has been proposed for certain cases, particularly in patients with significant comorbidities or slow-growing recurrences [4,13,17]. However, the risk of disease progression necessitates careful patient selection and shared decision-making between clinicians and patients.

Our findings aligned with previous literature (Table 4), emphasizing the importance of post-surgical surveillance and early intervention in high-risk patients. The management of LR should be tailored based on recurrence location and patient-specific factors. Surgical resection remains the preferred approach for resectable recurrences, while minimally invasive options like cryoablation and radiotherapy provide alternatives for patients who are poor surgical candidates. Systemic therapy is generally reserved for patients with high-risk disease or unresectable recurrence. When compared with previously published series, our cohort demonstrated comparable or slightly lower rates of LR and PSM despite a larger median tumor size (4.3 cm vs. 2.5–3.2 cm in other reports). Specifically, the median LR rate in our series was 1.3%, which is within the lower range reported in the literature (1.0–3.6%), while the PSM rate of 2.4% also compares favorably with prior studies (2.2–10%). The OS and CSS rates were slightly lower in our cohort (88.9% and 94.3%, respectively) than in some series, which may reflect differences in patient comorbidity profiles, tumor complexity, or follow-up duration. Taken together, these comparisons suggest that, even in larger or more anatomically complex tumors, meticulous surgical technique in experienced centers can achieve oncological outcomes consistent with published standards. While formal meta-analysis was beyond the scope of this study due to heterogeneity in follow-up and reporting, the integration of these data highlights the relevance of tumor characteristics, surgical approach, and center expertise in influencing recurrence and margin status.

The role of active surveillance in selected patients suggests that not all LRs require immediate intervention. Further research is needed to refine risk stratification models and optimize treatment algorithms.

An additional strength of our analysis was the minimal proportion of missing data (<5% across all variables), which not only reduced the risk of bias but also enhanced the internal validity and reliability of the reported associations. The high completeness of our dataset further supported the generalizability of these findings to similar surgical populations.

While our analysis suggested that the presence of positive surgical margins and certain histological variants may be associated with an increased risk of local recurrence, it is important to interpret these findings with caution. The number of recurrence events in our cohort was limited, resulting in wide confidence intervals that reduce the precision of the estimated associations. Consequently, these variables should be considered potential risk factors rather than definitive independent predictors of recurrence. Nevertheless, the observed trends were consistent with previously reported biological behavior of aggressive tumor subtypes and the recognized impact of incomplete resection, supporting their clinical relevance. Future studies with larger sample sizes and longer follow-up periods are warranted to confirm these associations and better quantify the magnitude of risk.

Although our study was not specifically designed to evaluate the impact of multidisciplinary care or center expertise on outcomes, several aspects of our findings indirectly support the importance of specialized management. All included patients were treated at high-volume centers with prospectively maintained registries, and surgeries were performed by experienced surgeons using advanced robotic or laparoscopic techniques. The overall low rates of positive surgical margins, local recurrence, and perioperative complications observed in our cohort likely reflect this concentrated expertise and coordinated perioperative management. These results suggested that outcomes in complex renal tumors are optimized when patients are managed in centers with multidisciplinary teams and surgical experience, emphasizing the potential benefit of specialized care even though this association was not formally tested in the present analysis.

A key limitation of the present study is that, while we provide a detailed description of treatment modalities applied at the time of initial local recurrence, we did not systematically capture outcomes such as progression-free survival, secondary recurrence rates, or long-term survival stratified by treatment strategy. Consequently, our data cannot directly answer the clinically relevant question of which modality—surgical resection, ablation, radiotherapy, or systemic therapy—offers the most durable oncological control after LR. This limitation reflects the retrospective nature of our dataset and the heterogeneity of management across centers. Nevertheless, the observation that nearly half of patients underwent surgical management, while a significant proportion received ablative or systemic therapies, underscores the real-world variability in LR treatment. Future prospective multicenter studies with standardized follow-up and uniform reporting of post-LR outcomes are needed to establish evidence-based recommendations on the optimal management strategy to improve prognosis after local recurrence.

In our analysis, histological variants were associated with an increased risk of local recurrence. For the purposes of this study, “variants” encompassed non–clear cell subtypes (papillary and chromophobe RCC) as well as cases exhibiting sarcomatoid or rhabdoid differentiation. However, due to the relatively small number of patients within each histological category, we were unable to perform a reliable stratified analysis of recurrence risk by specific subtype. This represents an important limitation, as sarcomatoid/rhabdoid differentiation is generally considered to confer a higher risk than papillary or chromophobe histology. Future multicenter studies with larger cohorts will be necessary to better delineate the differential impact of these distinct high-risk subtypes on recurrence patterns and prognosis, thereby enhancing the clinical applicability of histology-based risk stratification.

Our findings confirmed that renal function preservation after management of local recurrence remains achievable in the majority of patients, with outcomes consistent with published experiences. Repeat nephron-sparing procedures, despite their technical complexity, resulted in a modest but statistically significant decline in renal function, which aligns with prior multi-institutional analyses reporting median eGFR decreases of 7–10% following re-do partial nephrectomy [33,34]. Similarly, patients treated with ablative modalities demonstrated minimal renal function deterioration, corroborating long-term series showing negligible eGFR loss after cryoablation [35]. The subgroup receiving systemic therapy showed the greatest functional impairment, paralleling large randomized trials in advanced RCC where targeted therapies and immune checkpoint inhibitors were associated with nephrotoxicity and measurable eGFR decline [36]. Importantly, no patient in our cohort developed dialysis dependence, consistent with reports that nephron-sparing and ablative strategies rarely precipitate end-stage renal disease even in the setting of recurrence [34,35,36,37]. Taken together, our results highlight the importance of recurrence management strategies that prioritize renal preservation, confirming the superiority of nephron-sparing and minimally invasive approaches over systemic-only therapy when clinically feasible.

When stratified by treatment modality for local recurrence, our data demonstrated that surgical resection provides superior local control of recurrence compared to cryoablation or systemic therapy, while CSS and OS remain comparable between surgery and cryoablation in selected patients. These results mirror previously published series in which repeat partial nephrectomy was associated with recurrence-free survival rates exceeding 80% and durable cancer-specific survival [27,28]. Conversely, outcomes following ablative techniques, although associated with lower morbidity and better renal preservation, have shown higher recurrence rates, with 5-year LRFS ranging from 60% to 75% [29,30]. Systemic therapy alone yielded the poorest oncological outcomes in our cohort, consistent with randomized trials demonstrating inferior survival when systemic treatment is used without local control of disease [31]. Importantly, our findings reinforce the view that surgical resection remains the standard of care when technically feasible, while ablative approaches may be reserved for patients unfit for surgery or with limited renal reserve, in line with international guidelines [2].

We acknowledge limited power for several subgroup comparisons due to the small number of events in certain strata; this is reflected by wide confidence intervals for some univariable relative risks. Because of this imprecision, subgroup results should be interpreted as exploratory and hypothesis-generating rather than definitive. We mitigated this limitation by reporting absolute event counts, using exact tests for sparse data, and presenting adjusted effect estimates from multivariable logistic regression (which yielded more precise ORs). Larger multicenter series or pooled analyses are required to obtain precise risk estimates and to determine definitively the magnitude of risk across histological subtypes and margin status.

Although current international guidelines recommend structured postoperative surveillance after nephron-sparing surgery, our study adds nuance by evaluating real-world patterns of local recurrence (LR) and their timing. In our cohort, the median time to LR was 18 months, underscoring that the majority of recurrences occur within the first two to three years after surgery. This finding suggested that compliance with early follow-up is particularly critical. While our registries did not systematically capture modality-specific detection rates, both CT and MRI were used across centers, reflecting clinical practice; future research comparing the sensitivity of imaging techniques for early LR detection is warranted. Importantly, our results highlighted that certain subgroups—particularly patients with positive surgical margins or aggressive histological variants—demonstrated a higher risk of recurrence. These patients may benefit from shortened surveillance intervals or a tailored follow-up protocol, while standard intervals may remain appropriate for lower-risk patients. Thus, our findings support a more individualized, risk-adapted follow-up approach that goes beyond current uniform guideline recommendations and may improve the early detection of recurrence in high-risk patients.

## 5. Conclusions

LR after nephron-sparing or radical nephrectomy poses a significant clinical challenge, requiring careful follow-up and tailored treatment strategies. Although risk factors for recurrence are well-documented, they do not always predict outcomes, necessitating stringent radiological monitoring. Our findings indicate that positive surgical margins and histological variants are both associated with an increased risk of local recurrence after nephron-sparing surgery. Specifically, non–clear cell subtypes (papillary and chromophobe RCC) as well as sarcomatoid/rhabdoid differentiation emerged as potential high-risk categories. While these observations should be interpreted cautiously due to the limited number of events, they support the need for closer surveillance in such patients. Larger multicenter studies are warranted to validate histology- and margin-based risk stratification and to refine individualized follow-up strategies aimed at improving early detection and long-term oncological outcomes. Our findings highlight the need for a multidisciplinary, expert-driven approach to optimize patient outcomes and minimize disease progression. Future research should focus on refining predictive models and exploring novel therapeutic avenues for managing LR in RCC patients.

## Figures and Tables

**Table 1:** Clinical data.

Patients’ characteristics	
Age at diagnosis, years	64 (58–70)
Gender, m:f, %	73.3:26.7
ASA score, median (range)	2 (2–3)
AACCI, median (range)	4 (4–5)
Smoking status: not a smoker; an active/former smoker, %	50.4:49.6
eGFR, mL/min/1.73 m^2^, median (range)	67.4 (38–98)
Pre-operative tumor characteristics	
Focality: monofocal/multifocal, %	85.7:14.3
N° of lesions, median (range)	1 (1–2)
Median size of the largest lesion, cm (range)	4.2 (2.3–5.3)
Median RENAL score, median value (range)	7 (6–8)
Median Padua score, median (range)	7 (6–9)
Surgical characteristics	
Surgery: radical/partial nephrectomy	24.8:75.2
Approach: robotic/laparoscopic/open	59:32.4:8.6
Median length of surgery, minutes (range)	140 (120–182)
Median blood loss, mL (range)	200 (180–300)
Ischemia: no/yes, %	39.0:61.0
Median ischemia time, minutes (range)	21 (15.5–24)
Intraoperative complications, median rate, %	3.8
Perioperative complications, median rate, %	13.8
Pathological characteristics	
pTNM	
pT1a	43.5%
pT1b	26.3%
pT2	14.7%
pT3	15.5%
Histology,	
Clear cell RCC	84.0%
Papillary RCC	11.3%
Chromophobe RCC	3.7%
Variants/Aspects, %	10.5%
Median rate of LR, % (range)	1.3 (0.2–3.6)
Median time to LR, months (range)	18 (12–39)
Median rate of PSM, % (range)	2.4 (0–4.3)
OS, % (range)	88.9 (87.6–96.7)
CSS, % (range)	94.3 (89.9–98.4)

ASA: American Society of Anesthesiologists, AACCI: Adjusted Age-Adjusted Charlson Comorbidity Index, eGFR: estimated glomerular filtration rate, RENAL: Nephrometry Score, RCC: renal cell carcinoma, LR: local recurrence, PSM: positive surgical margin, OS: overall survival, CSS: cancer-specific survival.

**Table 4:** Comparison to other experiences (with complete data on tumor size, rate of LR, PSM, OS, and CSS).

	Tumor Size (cm)	LR (%)	PSM (%)	OS (%)	CSS (%)
Beauval et al. [11]	3.0	2.7	10.0	94.9	96.8
Bertolo et al. [12]	3.2	3.6	4.3	89.9	98.2
Khalifeh et al. [8]	2.9	1.0	2.2	88.0	99.2
Vartolomei at al. [29]	3.0	2.2	2.2	95.1	97.5
Chang et al. [30]	2.8	1.5	2.5	90.2	98.4
Alì Abdel Raheem et al. [31]	2.8	2.3	9.6	95.5	100
Kizilay et al. [32]	2.5	9.9	2.8	82.6	90.1
Our experience	4.3	1.3	2.4	88.9	94.3

LR: local recurrence, PSM: positive surgical margin, OS: overall survival, CSS: cancer-specific survival.

## Data Availability

The data presented in this study are available on request from the corresponding author.
